# Prevalence of thoracoabdominal imaging findings in tuberous sclerosis complex

**DOI:** 10.1186/s13023-022-02277-x

**Published:** 2022-03-15

**Authors:** David M. Ritter, Bailey K. Fessler, Daniel Ebrahimi-Fakhari, Jun Wei, David N. Franz, Darcy A. Krueger, Andrew T. Trout, Alexander J. Towbin

**Affiliations:** 1 Division of Neurology, Cincinnati Children’s Hospital Medical Center, Cincinnati, OH USA; 2 Department of Pediatrics, University of Cincinnati College of Medicine, Cincinnati, OH USA; 3 University of Cincinnati College of Medicine, Cincinnati, OH USA; 4 Department of General Pediatrics, University Children’s Hospital Muenster, Muenster, Germany; 5 The First Hospital of Yichang, Yichang, China; 6 Department of Radiology, Cincinnati Children’s Hospital Medical Center, Cincinnati, OH USA; 7 Department of Radiology, University of Cincinnati College of Medicine, Cincinnati, OH USA

**Keywords:** Tuberous sclerosis, *TSC2*, *TSC1*, MRI, CT, Renal angiomyolipomas, Renal cysts, Pulmonary nodules

## Abstract

**Background:**

Tuberous sclerosis complex (TSC) results in neurodevelopmental phenotypes, benign tumors, and cysts throughout the body. Recent studies show numerous rare findings in TSC. Guidelines suggest routine abdominal and chest imaging to monitor these thoracoabdominal findings, but imaging is not uniformly done across centers. Thus, the prevalence of many findings is unknown. To answer this, we categorized the clinical reads of 1398 thoracoabdominal scans from 649 patients of all ages in the Cincinnati Children’s Hospital TSC Repository Database.

**Results:**

Typical TSC findings were present in many patients: kidney cysts (72%), kidney fat-containing angiomyolipomas (51%), kidney lipid-poor angiomyolipomas (27%), liver angiomyolipomas (19%), and lung nodules thought to represent multifocal micronodular pneumocyte hyperplasia (MMPH) (18%). While many features were more common in *TSC2* patients, *TSC1* patients had a higher prevalence of MMPH than *TSC2* patients (24% versus 13%, p = 0.05). Many rare findings (e.g., lymphatic malformations and liver masses) are more common in TSC than in the general population. Additionally, most thoracoabdominal imaging findings increased with age except kidney cysts which decreased, with the 0–10 years age group having the highest percentage (69% 0–10 years, 49% 10–21 years, 48% 21 + years, p < 0.001). Finally, in our population, no patients had renal cell carcinoma found on abdominal imaging.

**Conclusions:**

These results show that regular thoracoabdominal scans in TSC may show several findings that should not be ignored or, conversely, over-reacted to when found in patients with TSC. Female sex, *TSC2* mutation, and age are risk factors for many thoracoabdominal findings. The data suggest novel interactions of genetic mutation with pulmonary nodules and age with renal cysts. Finally, in agreement with other works, these findings indicate that several rare thoracoabdominal imaging findings occur at higher rates in the TSC population than in the general population. This work supports obtaining detailed thoracoabdominal imaging in patients with TSC.

**Supplementary Information:**

The online version contains supplementary material available at 10.1186/s13023-022-02277-x.

## Background

Tuberous sclerosis complex (TSC) is a rare genetic disease resulting in neurodevelopmental disorders and benign tumors in multiple body organs with a highly variable phenotype [1–4]. TSC is an autosomal dominant disorder with a prevalence of 1 in 5000 to 1 in 10,000 people [5, 6] caused by germline mutations of *TSC1* or *TSC2*. Some patients have no mutation identified and are later discovered to have mosaicism in *TSC1* or *TSC2* [7]. *TSC1* and *TSC2* encode hamartin and tuberin which function to inhibit mTOR. In TSC, hamartin and tuberbin function is reduced allowing for overactivation of mTOR [8]. In phenotype-genotype correlations, patients with *TSC2* mutations tend to have a more severe phenotype compared to *TSC1* mutations [2, 4, 9, 10].

Patients with TSC are at high risk for autism and epilepsy and thus are frequently followed by pediatric neurologists [11]. Therefore, lesions outside of the nervous system have been slower to be characterized. Current guidelines suggest abdominal imaging every 1–3 years for all patients with TSC and females older than 18 should undergo additional screening for lymphangiomyomatosis (LAM) at least every 5 years with chest imaging [12]. However, clinical manifestations vary significantly from patient to patient ranging from no findings to multiple thoracic and abdominal lesions involving the heart, lungs, kidneys, liver, spleen, and pancreas [13].

Recently, the Tuberous SClerosis Registry to increase Awareness (TOSCA) study presented findings associated with TSC based on provider reports [11, 14]. In the TOSCA cohort, 17.3% of patients had a rare diagnosis and 2.9% had malignancies [14]. This, combined with thoracoabdominal findings of TSC reported by our group, highlights that while the focus of abdominal imaging is the renal manifestations of TSC, other organs need to be assessed [13]. Further, with the increasing use of mTOR inhibitors (mTORi) as disease-modifying therapy, manifestations of TSC beyond the hallmark manifestations of subependymal giant cell astrocytoma, angiomyolipomas, LAM, and epilepsy are increasingly relevant [15–21]. Indeed, rare findings such as pancreatic neuroendocrine tumors in TSC can also respond to mTORi treatment [22].

While there are multiple reports of these thoracoabdominal findings in TSC, there has been little systematic study of findings of TSC on imaging [2, 11, 14, 23]. TOSCA was able to look at diagnoses across numerous countries [11, 14]. While not all locations may be performing the same thoracoabdominal imaging surveillance, TOSCA found that the frequency of rare manifestations in TSC patients varied based on age, sex, and genetic mutation, with most findings more commonly occurring in adults, females, or patients with a *TSC2* mutation [14]. The lack of existing data and the links uncovered by TOSCA suggest that we need to understand the prevalence of thoracoabdominal imaging findings in TSC to (1) better understand genotype–phenotype associations, (2) assess risk for patients developing rare findings, and (3) determine best practices for surveillance and treatment of these rare findings associated with TSC. To address these issues, we performed a retrospective cohort analysis of the Cincinnati Children’s Hospital Medical Center (CCHMC) TSC repository database for the prevalence of thoracoabdominal imaging findings as determined by the clinical imaging reports of patients’ chest CTs, abdominal CTs, and abdominal MRIs.

## Methods

This was a retrospective cohort study of all patients enrolled in the Cincinnati Children’s Hospital TSC Repository Database with a confirmed clinical or genetic diagnosis of TSC who had thoracoabdominal imaging (CT, MRI) performed at CCHMC before July 1, 2019. The first and last imaging reports for MRI abdomen, CT abdomen, and CT chest were included for each patient to represent their disease course. This yielded 1398 thoracoabdominal scans from 649 patients of all ages. The earliest scan included in this dataset was from January 1997. Age, genetic mutation, and whether the patient had ever been prescribed a mTORi were additionally determined for each patient based on a review of electronic medical records. It is common practice in the CCHMC TSC Center, in agreement with guidelines, to obtain an MRI abdomen (or CT abdomen) every 2–3 years in all patients and a CT chest in females over the age of 18 every five years (as well as males with symptoms).

Clinical imaging reports were reviewed and specific findings were abstracted from the report. If the clinical read used non-definitive language regarding findings, two radiologists (ATT and AJT) reviewed the imaging to determine the presence or absence of results. If there was disagreement, a third radiologist served as a tie-breaker. Initial interpreting radiologists as well as reviewers for this study were aware of the patient’s diagnosis of TSC but were generally not aware of the patient’s specific genetic mutation unless that was included in the history provided for imaging (uncommon at our institution).

The percentage of patients with a thoracoabdominal finding in at least one of the two selected imaging examinations per patient was calculated. Percent of patients with these findings were categorized by sex (male/female) and genetic etiology. Genetic etiologies were broken down into mutations in *TSC1*, mutations in *TSC2*, patients with both *TSC1* and *TSC2* mutations (*TSC1/TSC2)*, no mutation identified in the patient (NMI), patients with reported positive testing but no results available at our institution (positive but unknown), and no testing done (not tested). Additionally, percentages of each finding were determined by binned age at the time of scan (0–10 years, 10–21 years, and 21 + years). Chi-square analysis was performed for the frequency of findings related to genetic mutation, age, and sex.

## Results

### Patient characteristics and imaging modalities used

**Fig. 1:**
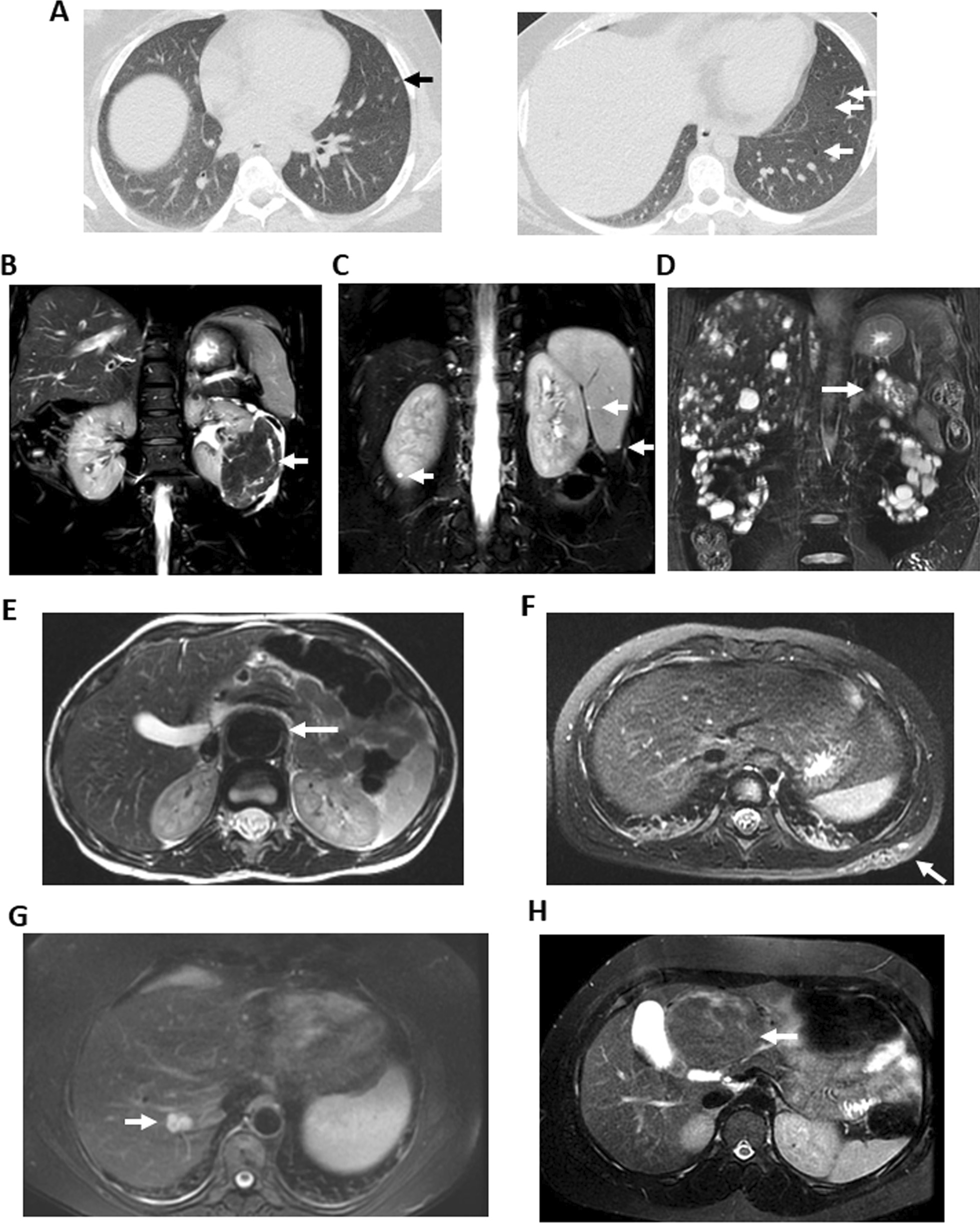
Representative imaging findings in TSC. **A** Axial CT chest in lung windows of 34 year old female with pulmonary nodules (black arrow) and cysts (white arrows). **B** 29 year old female with large AML arising from left kidney (arrow) on coronal T2-weighted fat saturated MRI. **C** 7 year old male with simple splenic and renal cysts (arrows) on axial T2-weighted fat saturated MRI. **D** 42 year old male with innumerable renal, hepatic, and pancreatic (arrow) cysts on coronal T2-weighted fat saturated MRI. Patient has had no genetic testing but has a clinically-suspected diagnosis of TSC2-PKD contiguous gene syndrome. **E** 5 year old male with abdominal aneurysm (arrow) on axial T2-weighted abdominal MRI. **F** 5 year old female with lymphatic malformation (arrow) on axial T2-weighted fat saturated MRI. **G** 53 year old female with hepatic hemangioma (venous malformation, arrow) on axial T2-weighted fat saturated abdominal MRI. **H** 15 year old female with hepatic adenoma (arrow) on axial T2-weighted fat saturated abdominal MRI

In our 649-patient cohort, there was an even split between sexes but, as consistent with prior studies and known epidemiology of TSC [9], more patients had identified mutations in *TSC2* than *TSC1* (45% versus 9%). Of the remainder with genetic testing performed, 1% identified mutations in both *TSC1* and *TSC2* (TSC1/TSC2), 1% of results were unavailable, and 9% had no identifiable mutation in either *TSC1* or *TSC2* (NMI, Table 1). In this cohort, 18 had TSC2-PKD1 contiguous gene syndrome, a large *TSC2* deletion, or a *TSC2* mutation without a report available to determine if they had contiguous gene syndrome. Abdominal imaging for the included patients was MRI abdomen only for 528 patients, CT abdomen only for 66 patients, and 22 patients with both MRI and CT abdomen. Of the 66 patients with CT abdomen only, 16 had vagal nerve stimulators as the likely reason for CT imaging. All chest imaging included was from chest CTs. As predicted from the TSC surveillance guidelines [12], more women had chest CTs than men (Table 1). The median age at time of scan was 17 years with a range of 0–77 years old. Representative imaging findings are shown (Fig. 1) but more detailed imaging characteristics have been previously published[13].

**Table 1:** Demographics

	Total	Male	Female
*By patient*			
All patients	649	306 (47%)	343 (53%)
Genetics			
TSC1	59	31 (53%)	28 (47%)
TSC2	289	137 (47%)	152 (53%)
NMI	59	31 (53%)	28 (47%)
TSC1/TSC2	8	6 (75%)	2 (25%)
Positive but unknown	9	6 (67%)	3 (33%)
Not tested	225	95 (42%)	130 (58%)
mTORi			
Yes	187	98 (52%)	89 (48%)
No	462	208 (45%)	254 (55%)
*By scan*			
All scans	1398	605 (43%)	793 (57%)
Imaging type			
CT abdomen	137	76 (55%)	61 (45%)
CT chest	280	53 (19%)	227 (81%)
MRI abdomen	981	476 (49%)	505 (51%)

### Manifestations of TSC apparent by thoracoabdominal imaging

Overall, 580 patients (89%) had findings on thoracoabdominal imaging. The most common thoracic findings overall were lung nodules thought to represent multifocal micronodular pneumocyte hyperplasia (MMPH) (18%), sclerotic bone lesions in the thoracic bones (14%), and lung cysts (12%) (Table 2, Fig. 1A). The most common abdominal findings were renal cysts (72%), renal angiomyolipomas (59% with any renal angiomyolipoma; 51% with lipid-rich angiomyolipomas, and 27% with lipid-poor angiomyolipomas), hepatic angiomyolipomas (19%), and sclerotic bone lesions in an abdominal or pelvic bones (15%) (Table 3, Fig. 1B–D). TSC patients are reported to be at increased risk for renal cell carcinoma [11, 24, 25]. However, no patient had imaging findings felt to be consistent with renal cell carcinoma in our cohort, even in patients > 40 years of age (N = 126).

**Table 2:** Thoracic findings in TSC patients grouped by sex and gene

	Lung cysts	Lung nodules	Lung effusions	Thoracic AML	Cardiac rhabdomyoma	Cardiac fatty focus	Cardiac aneurysms	Thoracic sclerotic bone lesion	Thoracic lymphatic malformation
Total (N = 649)	77 (12%)	119 (18%)	46 (7%)	8 (1%)	8 (1%)	38 (6%)	0 (0%)	90 (14%)	0 (0%)
*Sex, N (%)*									
Male (N = 306)	10 (3%)	29 (9%)	16 (5%)	2 (1%)	3 (1%)	12 (4%)	0 (0%)	14 (5%)	0 (0%)
Female (N = 343)	67 (20%)	90 (26%)	30 (9%)	6 (2%)	5 (1%)	26 (8%)	0 (0%)	76 (22%)	0 (0%)
*Gene, N (%)*									
*TSC1* (N = 59)	1 (2%)	14 (24%)	5 (8%)	0 (0%)	1 (2%)	2 (3%)	0 (0%)	7 (12%)	0 (0%)
*TSC2* (N = 289)	25 (9%)	39 (13%)	21 (7%)	4 (1%)	5 (2%)	17 (6%)	0 (0%)	36 (12%)	0 (0%)
NMI (N = 59)	3 (5%)	6 (10%)	1 (2%)	0 (0%)	0 (0%)	4 (7%)	0 (0%)	6 (10%)	0 (0%)
*TSC1/TSC2* (N = 8)	1 (13%)	2 (25%)	0 (0%)	0 (0%)	0 (0%)	1 (13%)	0 (0%)	1 (13%)	0 (0%)
Positive but unknown (N = 9)	2 (22%)	2 (22%)	0 (0%)	0 (0%)	0 (0%)	0 (0%)	0 (0%)	1 (11%)	0 (0%)
Not Tested (N = 225)	45 (20%)	56 (25%)	19 (8%)	4 (2%)	2 (1%)	14 (6%)	0 (0%)	39 (17%)	0 (0%)

*AML* angiomyolipoma

**Table 3:** Abdominal findings in TSC patients grouped by sex and gene

	Renal cysts	Renal fat-containing AML	Renal lipid-poor AML	Any renal AML	Renal cell carcinoma	Renal aneurysm	Hepatic cyst	Hepatic AML	Hepatic steatosis	Hepatic mass	Spleen hamartoma	Spleen cyst
Total (N = 649)	467 (72%)	334 (51%)	178 (27%)	385 (59%)	0 (0%)	8 (1%)	79 (12%)	125 (19%)	13 (2%)	8 (1%)	8 (1%)	23 (4%)
*Sex, N (%)*												
Male (N = 306)	225 (74%)	135 (44%)	82 (27%)	163 (53%)	0 (0%)	2 (1%)	31 (10%)	45 (15%)	5 (2%)	1 (0%)	3 (1%)	12 (4%)
Female (N = 343)	242 (71%)	199 (58%)	96 (28%)	222 (65%)	0 (0%)	6 (2%)	48 (14%)	80 (23%)	8 (2%)	7 (2%)	5 (1%)	11 (3%)
*Gene, N (%)*												
*TSC1* (N = 59)	33 (56%)	14 (25%)	6 (10%)	16 (27%)	0 (0%)	0 (0%)	5 (8%)	2 (3%)	4 (7%)	0 (0%)	0 (0%)	2 (3%)
*TSC2* (N = 289)	224 (78%)	128 (44%)	89 (31%)	156 (54%)	0 (0%)	2 (1%)	28 (10%)	48 (17%)	4 (1%)	3 (1%)	4 (1%)	8 (3%)
*NMI* (N = 59)	36 (61%)	28 (47%)	10 (17%)	30 (51%)	0 (0%)	0 (0%)	5 (8%)	3 (5%)	2 (3%)	1 (2%)	0 (0%)	2 (3%)
*TSC1/TSC2* (N = 8)	6 (75%)	3 (38%)	3 (38%)	4 (50%)	0 (0%)	0 (0%)	1 (13%)	1 (13%)	0 (0%)	0 (0%)	0 (0%)	1 (13%)
Positive but unknown (N = 9)	7 (78%)	5 (56%)	2 (22%)	6 (67%)	0 (0%)	0 (0%)	0 (0%)	2 (22%)	0 (0%)	0 (0%)	0 (0%)	1 (11%)
Not Tested (N = 225)	161 (72%)	156 (69%)	68 (30%)	173 (77%)	0 (0%)	6 (3%)	40 (18%)	69 (31%)	3 (1%)	4 (2%)	4 (2%)	9 (4%)

	Adrenal AML	Adrenal adenoma	Pancreatic cyst	Pancreatic AML	Pancreas neuroendocrine tumor	Abdomen sclerotic bone lesion	Abdominal aneurysm	Abdominal vascular stenosis	Abdominal lymphatic malformation	Abdominal PEComa
Total (N = 649)	8 (1%)	3 (0%)	13 (2%)	3 (0%)	7 (1%)	98 (15%)	5 (1%)	2 (0%)	8 (1%)	1 (0%)
*Sex, N (%)*										
Male (N = 306)	3 (1%)	0 (0%)	5 (2%)	1 (0%)	5 (2%)	35 (11%)	2 (1%)	0 (0)%)	0 (0)%)	0 (0)%)
Female (N = 343)	5 (1%)	3 (1%)	8 (2%)	2 (1%)	2 (1%)	63 (18%)	3 (1%)	2 (1%)	8 (2%)	1 (0%)
*Gene, N (%)*										
TSC1 (N = 59)	1 (2%)	1 (2%)	0 (0%)	0 (0%)	1 (2%)	8 (14%)	1 (2%)	0 (0%)	0 (0%)	0 (0%)
TSC2 (N = 289)	2 (1%)	1 (0%)	8 (3%)	0 (0%)	2 (1%)	32 (11%)	2 (1%)	2 (1%)	5 (2%)	1 (0%)
NMI (N = 59)	0 (0%)	0 (0%)	1 (2%)	0 (0%)	0 (0%)	6 (10%)	1 (2%)	0 (0%)	0 (0%)	0 (0%)
TSC1/TSC2 (N = 8)	0 (0%)	0 (0%)	0 (0%)	0 (0%)	0 (0%)	0 (0%)	0 (0%)	0 (0%)	0 (0%)	0 (0%)
Positive but unknown (N = 9)	0 (0%)	0 (0%)	0 (0%)	0 (0%)	0 (0%)	0 (0%)	0 (0%)	0 (0%)	0 (0%)	0 (0%)
Not Tested (N = 225)	5 (2%)	1 (0%)	4 (2%)	3 (1%)	4 (2%)	52 (23%)	1 (0%)	0 (0%)	3 (1%)	0 (0%)

*AML* angiomyolipma, *PEComa* Perivascular epithelioid cell tumor

### mTOR inhibitor use

Our study cohort spans the time where mTORi became used more frequently in TSC. In our cohort, patients from all ages were equally likely to be prescribed mTORi (percent prescribed mTORi: 0–10 years = 33%, 10–21 years old = 37%, > 21 years old 30%). Those prescribed an mTORi had a higher prevelence of typical TSC findings: renal cysts, renal fat-containing AMLs, renal lipid-poor AMLs, hepatic AMLs, and lung nodules (Table 4).

**Table 4:** Frequent findings in TSC patients based on mTORi prescription

	Kidney cysts	Kidney fat-containing AML	Kidney lipid-poor AML	Liver AML	Lung cysts	Lung nodules
mTORi prescribed (N = 186)	146 (79%)	110 (59%)	60 (32%)	41 (22%)	21 (11%)	40 (22%)
mTORi not prescribed (N = 463)	320 (69%)	224 (48%)	117 (25%)	83 (18%)	56 (12%)	79 (17%)

*AML* angiomyolipoma, *mTORi* mTOR inhibitor

### Sex differences

Thoracic findings overall were more common in females (Table 2), primarily because women were four times more likely to have chest imaging than men (Table 1). However, when only looking at those patients who received chest CTs, lung cysts and thoracic sclerotic bone lesions were still more common in women (Additional file 1: Table S1). Lung cysts and nodules were most prevalent, consistent with early or definite LAM and MMPH that is known to occur in adult women with TSC [26]. Most males (84%) had no CT imaging of the chest, as asymptomatic males do not require LAM surveillance [12, 27, 28]. However, 60% of men with a chest CT showed pulmonary nodules and 20% had pulmonary cysts (Additional file 1: Table S1), suggesting that men also are at risk for pulmonary involvement even if overall prevalence is much lower than that reported for females (Table 2). Female patients had a higher prevalence than male patients of most abdominal findings including kidney angiomyolipomas, liver angiomyolipomas, sclerotic bone lesions, and lymphatic malformations (Table 3). Furthermore, while multiple female patients had an abdominal lymphatic malformation or an adrenal adenoma, no male had these findings on abdominal imaging.

### Genetic differences

Supporting the understanding that patients with *TSC2* mutations have a more severe phenotype [2, 4, 9], most imaging findings were more prevalent in this group (Tables 2 and 3). Specifically, there is a fourfold increase in lung cysts (χ^2^ = 4.14 p = 0.04), a twofold increase in renal angiomyolipomas (χ^2^ = 14.14 p < 0.001), and a fivefold increase in liver angiomyolipomas (χ^2^ = 6.96 p = 0.008) if the patient has a *TSC2* mutation compared to *TSC1* mutation. Conversely, *TSC1* patients were more likely to have lung nodules than *TSC2* patients (χ^2^ = 3.97, p = 0.05, Table 2).

### Age differences

All thoracoabdominal manifestations of TSC increased with age except for kidney cysts, which paradoxically decreased with age (χ^2^ = 42.39, p < 0.001, Table 5).

**Table 5:** Most frequent thoracoabdominal findings found in TSC patients grouped by age

	Kidney cysts	Kidney Fat-containing AML	Kidney lipid-poor AML	Any AML	Liver AML	Lung nodules	Abdominal sclerotic bone lesion	Thoracic sclerotic bone lesion	Liver cyst	Lung cysts	Lung effusions
0–10 years (N = 432)	296 (69%)	78 (18%)	58 (13%)	122 (28%)	9 (2%)	3 (1%)	7 (2%)	0 (0%)	9 (2%)	0 (0%)	15 (3%)
10–21 years (N = 416)	202 (49%)	201 (48%)	67 (16%)	228 (55%)	60 (14%)	46 (11%)	33 (8%)	33 (8%)	4 (1%)	16 (4%)	11 (3%)
21 + years (N = 550)	265 (48%)	351 (64%)	112 (20%)	383 (70%)	145 (26%)	109 (20%)	81 (15%)	74 (13%)	84 (15%)	104 (19%)	28 (5%)

### Rare findings

In our cohort, eight patients had abdominal lymphatic malformations, eight had renal arterial aneurysms, five had abdominal aneurysms, and two had vascular stenosis (see Fig. 1E, F). These were detected even though vascular imaging-specific sequences are not routinely done on patients with TSC.

While renal lesions predominate in TSC and are the primary reason serial abdominal imaging is recommended in TSC, hepatic lesions were a frequent finding (Fig. 1G, H). Specifically, hepatic masses (5 without a specific read by the radiologist but differential included benign or malignant tumors, 1 read as likely follicular nodular hyperplasia, 1 read as hepatocellular neoplasm, and 1 read as hepatic hemangioma) were found more frequently than reported in the general population [29]. Biopsy of one patient’s liver lesion showed hepatic adenoma.

Cysts were also identified in the liver, spleen, and pancreas of patients with TSC (Table 3). Cysts were found in patients not just with polycystic kidney disease or TSC2-PKD1 contiguous gene deletion syndrome. In those with liver cysts, 26/80 had no renal cysts or a *TSC1* mutation. Meanwhile only 2/23 of the splenic cysts patients had clinically diagnosed contiguous gene syndrome (no confirmatory testing, see example in Fig. 1D) and 12/23 patients were genetically confirmed not to have TSC2-PKD1 contiguous gene syndrome (see example in Fig. 1C).

## Discussion

Here we report the prevalence of thoracoabdominal imaging findings in a retrospective cohort of TSC patients spanning 20 years of clinical surveillance. The data add to the knowledge of TSC features observed throughout the patient’s lifespan. Our results suggest, with rare exceptions, that female sex, *TSC2* mutation, and increasing age contribute to the risk of developing TSC-related thoracoabdominal findings. Additionally, several findings, including aneurysms and hepatic masses, occur at higher frequencies than expected in the general population, reflecting possible novel contributions to the TSC phenotype. Our results strongly support the recommendation for detailed imaging of the entire abdomen, rather than alternative modalities such as ultrasound focused on kidneys alone, as the International TSC Consensus Group reaffirmed in 2021 for guiding treatment decision-making and monitoring responses [12].

Previous work in other TSC cohorts has yielded similar results to our data [11, 14, 23], such as the prevalence of renal angiomyolipomas (40–60%) [11, 23]. Similarly, the prevalence of pulmonary cysts, a diagnostic hallmark of LAM, was consistent with previous estimates of LAM in TSC (7–28%) [11, 23, 26]. This is reassuring given the differences in the composition of the study cohort (large, long-standing single-center multidisciplinary TSC clinic vs. multicenter cross-sectional studies) and ascertainment methodology (direct imaging reports vs. clinician-supplied diagnoses). Additionally, the prevalence of findings in our older age group agrees with those in a Spanish TSC adult cohort [23].

There are, however, some interesting differences between our work and others. We found a higher prevalence of renal cysts in patients (72% compared to 23–44%, [11, 23]), which we suspect can be attributable to the fact that cystic renal disease is a minor diagnostic criterion in TSC, and therefore may be overlooked when relying solely on clinician (or patient) reporting without verification through direct assessment of the actual images or radiologist’s reported findings. Additionally, this might depend on the type of imaging modality used in previous studies (MRI versus CT versus ultrasound). For example, within our repository only 17 patients were not included in this cohort because they only had renal ultrasounds. Of those, 9 ultrasounds were read as normal, 7 had renal AMLs, 1 had renal cysts, and 2 had other findings (nephromegaly and “nonspecific lesion”). Importantly, 2 ultrasounds read as normal had subsequent MRIs after our study inclusion cutoff which showed renal cysts. Another difference is the identification of sclerotic bone lesions and cardiac rhabdomyomas, which can partly be explained by the imaging modalities utilized. MRI is recommended for abdominal surveillance rather than CT or ultrasound in TSC due to the improved visualization of renal angiomyolipoma on MRI, especially when lesions contain little or no fat [30]. While sclerotic bone lesions would be more readily appreciated with CT, renal angiomyolipoma represent a greater risk for clinical complications and MRI utilization should continue to be prioritized over CT for this reason [12]. Cardiac rhabdomyomas are best visualized by cardiac echocardiogram, although dedicated cardiac MRI approaches exist for high-resolution detection of rhabdomyomas and other structural cardiac abnormalities [31].

Surprisingly, we had no imaging diagnosis of renal cell carcinoma even though this has been associated with TSC [11, 24, 25]. Within our cohort there were 3 renal biopsies done at CCHMC. These were done after renal transplant related to TSC2-PKD1 contiguous gene syndrome and not for concerns of renal cell carcinoma. We are unsure why the radiologists do not suggest renal cell carcinoma but there are several possibilities including frequent imaging at our instituation to allow for comparison imaging, the use of MRI as a predominate imaging modality (Table 1), influence of mTORi treatment, or a bias in the images included. While imperfect to suggest this is not a biased cohort, no patient had a diagnosis of renal cell carcinoma in the electronic medical record to suggest that they received treatment elsewhere for renal cell carcinoma. Multicenter studies looking at repeated MRI imaging from TSC patients, pathology-based diagnoses, and treatment approaches are needed to understand this finding.

In agreement with prior work [14], we found several rare findings with a higher prevalence in our cohort than reported in the general population. Specifically, liver masses as a group were found in 1% of TSC patients, although in the general population it is 1 in 100,000 to 1 in 1,000,000 [29]. Pathologic diagnosis to identify these masses will help determine the relation between TSC and liver lesions. We also found lymphatic malformations in the abdomen at 1–2% based on the patient’s risk factors (female sex and *TSC2* mutation). This is consistent with the already known association of congenital lymphatic problems and TSC [32, 33]. Additionally, cysts within multiple abdominal organs were found, regardless of the status of TSC2-PKD1 contiguous gene syndrome diagnosis, suggesting that this could be the result of TSC-specific pathways. However, not all rare imaging findings may be TSC-related. Adrenal adenomas were rare in our TSC cohort, occurring at a frequency similar to those detected incidentally in the general population 0.5–2% [34].

Many of our findings were not felt to be clinically significant by the clinical team and thus biopsies or further tissue work up has not been pursued. Thus, the direct association with TSC has not been proven. Further work looking at histopathology, incidence of imaging findings in the general population, and the effect of mTORi treatment will help in our understanding. Most of these findings are too rare to include as part of the TSC syndrome diagnostic criteria but knowledge of these rare findings found on screening MRIs and CTs should allow radiologists and TSC treating physicians to better discuss treatment implications, subspecialty referral, and follow up imaging. Furthermore, routine MRIs in children instead of renal ultrasounds found these lesions that occur at a higher incidence than the population allowing for interventions if needed before serious complications (see Fig. 1E, F, and H).

It has previously been reported that females are more likely to have rare findings in TSC and particularly have higher rates of LAM [14, 28] and congenital lymphatic disease [33], consistent with our results. Based on LAM surveillance imaging recommendations [12], females are more likely to have chest imaging, but abdominal imaging was performed equally across sexes (Table 1). Estrogen is thought to contribute to the risk for LAM [12, 28], but it remains unclear if this or other factors influence the prevalence of other TSC manifestations. The link between estrogen and LAM is also thought to explain why most male patients with TSC do not develop LAM and therefore LAM surveillance imaging is not routinely performed in asymptomatic males [12]. Consistent with these prior observations and clinical practice, males in our cohort were less likely to have cysts and nodules. We cannot say definitively if the nodules reported are due to MMPH, which is associated with TSC, or other causes such as past infections, smoking, or other chronic health issues [35]. The latter could explain our observation that pulmonary nodules did not exhibit a preference for TSC patients with a mutation of *TSC2,* unlike most other identified radiological findings in our study. Pulmonary nodules were more likely to be found in patients with mutation of *TSC1* rather than *TSC2*. We cannot explain this observation and more analysis would be needed to confirm this finding and investigate the underlying mechanisms involved, especially as that difference appears less when looking only at patients who received chest CTs (Additional file 1: Table S1). Regardless, the presence of lung nodules and lung effusions should remind TSC providers that lung findings in TSC are not exclusive to females and to not ignore the possibility of pulmonary TSC manifestations in males or seemingly less affected patients with *TSC1* mutations.

Nearly all thoracoabdominal findings increased with age (Table 5). This is not surprising as many imaging findings such as angiomyolipomas and LAM are routinely not seen until the second or third decade of life [2, 11, 12, 28]. However, we found that renal cyst detection decreased with age. Others have reported small studies suggesting that younger patients had more renal cysts detected [36]. It is possible that this is the natural history of TSC renal cysts or that the increasing use of mTOR inhibitors has decreased the prevalence of renal cysts in the modern era [37]. In our cohort we only have whether patients were prescribed mTORi or not. As a descrete variable, mTORi treatment did not detect any difference in renal cysts (Table 4) but this is likely to indicate that patients with more disease burden were more likely to be on mTORis. Systematic study of cystic renal disease in TSC is needed and an emerging area of active research to better understand its underlying pathogenesis and natural history in TSC [38].

We do acknowledge our study has limitations. It is a retrospective cohort at a single United States TSC center. As such, the clinical decision-making for obtaining imaging and the frequency thereof, imaging technologies available and corresponding acquisition protocols utilized, and the interpretation of imaging results are institution-specific. Also, the current study analyzed the radiologist’s reported findings rather than re-evaluation and scoring of the images themselves. Caution, therefore, must be given as images for each patient were not re-reviewed. Thus some findings may be missed or subject to the variability of radiologists’ skill or reporting practices. Furthermore, many patients may have imaging performed at other institutions not included here, possibly altering the prevalence of specific findings. We did not include ultrasound imaging as it does not cover the entire abdomen or chest and thus any unique findings on ultrasound are not counted in this cohort. Finally, we did not track all scans from an individual patient to see how their results changed, whether mTORi impacted findings on subsequent scans, or if reported findings were consistently interpreted over time.

## Conclusion

These data provide prevalence estimates of thoracoabdominal findings in a cohort of TSC patients receiving routine screening imaging of the abdomen and chest. Knowledge of these findings and their prevalence over time will help TSC specialists and radiologists optimize the frequency of imaging to monitor clinical disease and increase awareness of rare imaging findings discovered on imaging. Our data suggest that female sex, age, and *TSC2* mutations are associated with a higher risk for most findings except pulmonary nodules and renal cysts. Future work is needed to understand the disease progression of abdominal and thoracic TSC manifestations, determine which manifestations require intervention, and produce best practices for thoracoabdominal imaging in TSC. Our data, along with prior work, supports the use of routine, detailed thoracoabdominal in TSC patients.

## Supplementary Information

**Additional file 1**. Thoracic findings in TSC patients recieving a chest CT.

## Data Availability

All data generated and analyzed during this study are available in the published manuscript.

## Abbreviations

CCHMC: Cincinnati Children’s Hospital Medical Center
LAM: Lymphangiomyomatosis
mTORi: MTOR inhibitor
NMI: No mutation identified
TSC: Tuberous sclerosis complex
TOSCA: Tuberous SClerosis Registry to increase Awareness
