# Supplementary material for: Prevalence of thoracoabdominal imaging findings in tuberous sclerosis complex
Source: Orphanet J Rare Dis. 2022 Mar 15;17:124. doi: 10.1186/s13023-022-02277-x (PMC8922878; doi:10.1186/s13023-022-02277-x)
Supplement: Supplementary file 1 — Additional file 1. Thoracic findings in TSC patients recieving a chest CT. [file 13023_2022_2277_MOESM1_ESM.docx]

**Table S1: Thoracic Findings in TSC patients receiving a chest CT**

|  | **Lung Cysts** | **Lung Nodules** | **Lung Effusions** | **Thoracic AML** | **Cardiac Rhabdomyoma** | **Cardiac Fatty Focus** | **Cardiac Aneurysms** | **Thoracic Sclerotic Bone Lesion** | **Thoracic Lymphatic Malformation** |
| --- | --- | --- | --- | --- | --- | --- | --- | --- | --- |
| **Total (N= 201)** | 77 (38%) | 119 (59%) | 46 (23%) | 8 (4%) | 8 (4%) | 38 (19%) | 0 (0%) | 90 (45%) | 0 (0%) |
| **Sex, N (%)** |  |  |  |  |  |  |  |  |  |
| **Male (N=48)** | 10 (21%) | 29 (60%) | 16 (33%) | 2 (4%) | 3 (6%) | 12 (25%) | 0 (0%) | 14 (29%) | 0 (0%) |
| **Female (N=153)** | 67 (44%) | 90 (59%) | 30 (20%) | 6 (4%) | 5 (3%) | 26 (17%) | 0 (0%) | 74 (48%) | 0 (0%) |
| **Gene, N (%)** |  |  |  |  |  |  |  |  |  |
| **TSC1 (N=20)** | 1 (5%) | 14 (70%) | 5 (25%) | 0 (0%) | 1 (5%) | 2 (10%) | 0 (0%) | 7 (35%) | 0 (0%) |
| **TSC2 (N=71)** | 25 (35%) | 39 (55%) | 21 (30%) | 4 (6%) | 5 (7%) | 17 (24%) | 0 (0%) | 36 (51%) | 0 (0%) |
